# The Treatment of Phthisis Pulmonalis with Watery Extract of Tubercle Bacilli*Read at thirty-second annual meeting of the Texas State Medical Association, Waco, Texas, April 26, 1900.

**Published:** 1900-08

**Authors:** James D. Osborne

**Affiliations:** Cleburne, Texas


### TEXAS MEDICAL JOURNAL.

ESTABLISHED JULY, 1885.

PUBLISHED MONTHLY.—SUBSCRIPTION $1.00 A YEAR.

VOL. XVI. AUSTIN, AUGUST, 1900. No. 2.

### Original Contributions.

For the Texas Medical Journal.

### The Treatment of Phthisis Pulmonalis with Watery Extract of Tubercle Bacilli.*

BY JAMES D. OSBORNE, M. D., CLEBURNE, TEXAS.

In the outset, please remember that nothing original is claimed in this article, and that it is only a slight testimonial to those learned delvers in the laboratories, where, with crucibles, retorts, reagents, and microscopes, they are wearing their lives out for the betterment of mankind, and battling to overcome the greatest of human ills, consumption.

Medical science and the advance in civilization have practically stamped out the plague, smallpox, typhus fever, and they hold yellow fever and cholera at bay. They are steadily and surely gaining headway in the crusade against the greater foe, consumption. In 1882, Dr. Robert Koch made the startling announcement that this fell destroyer was caused by the introduction of a living germ—the *bacillus tuberculosis*—into the system of a previously healthy person. Scientific men all over the civilized world were enthused, startled, and took up the study on the lines indicated by Koch, with the result that his theories were verified and accepted; and today the fact is surely established that tuberculosis is a communicable and

*Read at thirty-second annual meeting of the Texas State Medical Association, Waco, Texas, April 26, 1900.

preventable disease, and that the only way by which a healthy person or animal can contract it is by means of a living germ, which grows and propagates its kind in the body of man or animal.

This discovery tore aside the veil of mystery, doubt, and uncertaincy, and cast into outer darkness the false doctrines and theories which had surrounded the theories of tuberculosis; and now this little living germ is acknowledged as the sole cause of this most fatal and dreaded scourge of the human race. Farther than this, Koch's discovery has proved beyond dispute that nobody does, nor can, inherit consumption. It has cast aside the idea of physical predestination, from which there was no escape, and no such taint is inherited from birth. Consumptive ancestors do transmit to their offspring a predisposition to infection from which they themselves suffer—that is, the children inherit a physical organism which is deficient in power to resist the attack of this particular germ, if once it receives an entrance into the system.

The offspring of consumptives must first receive infection from without before they acquire consumption. These differ from others in this way: once they are infected, the disease is more rapid, and their constitution less liable to throw off the disease. Then, to members of consumptive families we can offer new hope, because the discovery of this little germ teaches us that to avoid the disease it is only necessary to avoid being infected. This discovery of Koch's enables us to carry on an effective and intelligent warfare against this prolific germ all over the world. Thus we can reduce the death rate from consumption and tubercular affections; and by united and combined efforts and with the intelligent army of medical men and educated civilization, we can stamp out the disease by the extinction and extermination of the tubercle bacilli.

Following up Koch's theory, we are indebted to such men as Fisch, Edson, Mariglioni, and Von Ruck for great practical researches and the constant warfare against this insidious foe. Following these lines for the past four years, with greedy desire, I have been battling with the germs, and have used all their methods and treatments, and with varying results. At one time my hopes would run high, and again be dashed miserably against the rocks of despair. With the consumptive patient you certainly have a willing subject: there is no pain or suffering from a hypodermic injection of aseptolin, Mariglioni's serum, of Fisch's antiphthisic serum, or Von Ruck's watery extract of tubercle bacilli, and neither will cause a halt or murmur of dissent; for they are helping you to stamp out and kill the germ.

From Edison's treatment with aseptolin, I think I had the best results, until my attention was directed, in the summer of 1899, to Von Ruck's treatment of tuberculosis with the watery extract of tubercle bacilli. Dr. Von Ruck, an eminent German physician, has built up and established in the great health resort, at Asheville, in the mountains of North Carolina, the Winyah Sanitarium and Laboratory, where he manufactures his watery extracts of tubercle bacilli, and is successfully treating consumptives. I have never met him; but my correspondence with him, and the meeting of personal friends of his, led me to believe that he is a conscientious scientific worker; and now, you might say, with personal knowledge of the discoverer of the watery extract of tubercle bacilli, I have not hesitated to employ his methods, and to this time with more satisfactory results than from all the others combined. It certainly has one great element in its favor; it produces no pain except the pricking of the hypodermic needle, and he recommends, and I use, with each case, a separate, sharp, minute needle, sterilizing it before and after using.

If the patient be not too far advanced in consumption—that is, if in the first stage, we can and do hold out the promise that a cure can be effected. In cases where nutrition is bad, cavities formed, and prostration great, amaciation going on, chest sinking in, and night sweats exhausting, there is no use in using the remedy; for it is impossible for nature to overcome the great loss of lung tissue, weakening and inanition; a prostrated "lunger" has no hope and it is wrong to place such a case among those amenable to treatment.

Dr. Von Ruck recommends and advises this treatment chiefly in the early and middle stages, free from absorption fever, with a fair degree of nutrition, and free from serious complications; from such cases good results can be promised, and after a few weeks treatment you can easily detect changes; for percussion and auscultation show in the tubercular area a weak, feeble respiratory murmur with a general physical improvement. Under continued treatment, the physical symptoms, due to fibroid changes, caseous pneumonia, thickening of pleura, and such like, clear up and disappear. All this improvement is gradual and slow; but you will note a gradual improvement; hope becomes buoyant, breathing easier, cough moderating, and a steady gain in flesh; a natural eye, stronger voice, and better appetite, and sounder sleep, and a more elastic step—all harbingers that the removal of non-degenerative tubercle will follow, and new advances and the degenerative and the destructive process will be controlled. Bring to your aid now the nutritive and reparative processes of the system, and the tubercular infiltration is an accomplished fact. Of course, it is a tedious treatment, taking month and even years to complete. After making your diagnosis, your patient understanding all the array of troubles and trials that will have to be undergone, and with the promise of faithful compliance to every detail—to diet and fresh air, you are ready to begin.

We have eight cases under treatment and observation, and all show good results. We commence the treatment by using antiseptic washes and sprays to the larynx, mouth, fauces, teeth, daily for the first three or five weeks, and if any catarrh, which is rarely absent, it must be treated in the same manner, and then commence with a subcutaneous injection of one-tenth of one cubic centimeter of No. 1 of the watery extract of tubercle, and increase every day by one-tenth cubic centimeter, until the daily dose is one cubic centimeter. The No. 1 watery extract contains 1/100 of one per cent. of the solid anhydrous extract of tubercle bacilli. No. 10 contains 1/10 of one per cent. of the extract. No. 100 contains one per cent. of the extract. On account of the bulk of the dose after you have reached one cubic centimeter, it is best to use No. 10, and of this we begin with 1/10 of one cubic centimeter, and increase by tenths, as one-tenth of this is equal to one cubic centimeter of No. 1; increase with this until one-half of one cubic centimeter is reached. From now on, we find that this dose is quite active, and note a perceptible influence over the tubercular processes. The increase in dose from now on is more gradual, and as the doses are larger the intervals may be extended from twenty-four to forty-eight hours; then it is best to commence on No. 100, and begin with 1/10 cubic centimeter, which is equal to one cubic centimeter of No. 10, or ten cubic centimeters of No. 1. The reactions are often so marked that we desist, and let our patients rest for several days, or, until the reactive fever subsides. Very little irritation follows hypodermic injection. If any should follow, it will disappear in about 24 to 30 hours; and as the treatment is followed up, toleration is established. This is one great advantage over the aseptolin treatment, which will not bear toleration. The pain from the injection of the medicine growing greater from every injection, and the bulk of the dose make serious objection.

The best site for the subcutaneous injection I have found to be in the intra-scapular region, always using caution at the point of injection by rendering the parts perfectly aseptic, and make no second injection at the same point for a week or ten days.

Here, I wish to present the report of a case that was far advanced in its second stage. I read the letter from the patient himself, as

I requested him to give it to me in his own language and in his own ideas. He is a tobacco fiend and self-willed, and will not give up the use of it. His cure is simply wonderful. He does not tell you how much lung tissue was involved; but I tell you both apices were involved, the claviculous spaces sunken, laryngitis characteristic, emaciation complete, night sweats exhausting, and it looked as if death was near.

March 10, 1900.

DEAR DOCTOR:—Your letter received, and I hasten the reply. I hardly know where to begin. In 1890, I had the grip, which was my starting point. Prior to that time I weighed 160 pounds; was in good health. I run down in flesh as low as 110. In 1893, examination of sputa showed bacilli germs in large quantities; had depression and pain in my left lung; pulse weak; general condition very poor. Had fever, night sweats, cough, etc. This continued with varied degrees until 1898, when I commenced this treatment. At no time, for any length of time, during the period from 1893 to 1898, was I free from fever or expectoration of large quantities of thick purulent matter; always felt debilitated, nervous, and unfit for any duty that required the least exertion or close study. In June, 1899, had a most severe hemorrhage; the worst I ever had,—having had four or five prior to that time. On June 29, 1899, I began taking the treatment. Took it every day. The doses were increased some every day until full dose was reached. At the time I commenced the treatment I was in the worst shape I have ever been. It was shortly after my severe hemorrhage, and I had run down in weight to 110 pounds, coughed continually, and expectorated a heavy yellow pus; in the morning when I would wake, the moment I stood on my feet, large quantities of it that had accumulated during the night would be thrown off. Had fever, pain in the chest, depression, difficulty in breathing, weak heart's action. I felt better as the treatment progressed. Today I weigh 128½ pounds; breathing good, pulse strong, appetite excellent, not worried by exercise, attend to my office duties, and upon examination later, my lungs tested, found breathing well and full, no rattling, almost free from coughing, not so susceptible to cold as formerly; in fact, I feel good. Am thoroughly pleased with the treatment, and the first of next month I want to take another bottle of No. 10 watery extract, tone up, and put on the finishing touches.

Another thing, doctor, I believe the treatment has kept me free from the grippe this winter, as it is the first time I have missed the grippe in ten years. After the first bottle of medicine, the fever left me, and I continued to improve all along. My nerves began to settle, and I felt stronger each day. My weight would be more now, I feel satisfied, if I had followed your instructions about tobacco; but I just can't quit it. My digestion has improved wonderfully.

Now, not being a doctor, I don't know if I have answered along the lines you wish; but, if not, anything you suggest, I will try and answer."

Since the profession is recognizing the fact that tuberculosis is much more prevalent in a latent or not very active form, it behooves us to make an early diagnosis; and all the scientific observers now know that the tuberculin test will discover the presence of the disease even in its incipient stage; because, when injected under the skin of a person free from tuberculosis, it produces no symptoms; but, if the disease is present in the slightest degree, the injection causes fever and other characteristic symptoms.

You can safely rely on a sensitive response through the injection of the watery extract of tubercle bacilli, and we do not wait for the microscope to reveal to us the germ. But for several days after the diagnosis, we keep up a diagnostic treatment, and keep an accurate record of temperature and changes. For the commencing days, we use from one to three-tenth cubic centimeters of the Watery Extract, according to the age and sex, and double this dose in thirty-six and forty-eight hours, until we see whether we get the reaction. After that we wait a day or two and double the dose again. If no reaction has occurred after four injections, then we are convinced that we have no pre-tubercular troubles to contend with, and a negative diagnosis is established. This reaction may be shown in a rise of temperature of from one to five degrees. We cause the subject to take the temperature for forty-eight hours before using the test. About three or four hours after this, we inject and cause some observations of temperature to be made. Of course, the higher the fever of reaction runs the greater degree of infection. Rgarding every case of a chronic nature or obscure origin as tubercular, it becomes our duty to exclude that idea by a thorough test with the extract. The diagnosis may have been made from a pulmonary hemorrhage, laryngitis, an old pleurisy, a daily rise of temperature with a quickened pulse, bronchial catarrh, impaired digestion, enlarged glands, diseased tonsils, or from some pathological condition of the mucous, serous, and synovial membranes; but you are still uncertain. The presence of tubercular germs is surely pathognomonic. Nothing else can make you positive. Then bring to your aid the positive test with the watery extract of tubercle bacilli, which brings to our knowledge their true nature with gratifying certainty.

As we have learned the very important and practical knowledge, that by the gradual introduction into the blood of the toxines from certain pathogenic micro-organisms, immunity can be established, so that the growth and multiplication of these germs can no longer continue in the animal or person so treated, why not hold out to those who have been by any means subjected to infection the promise of certain relief from the disease by immunization? I doubt not the time will soon come when it can be absolutely prevented. In this manner consumption will be stamped out.

I am closely watching two cases that are infected, and have immunized them; and so certain am I of this treatment that in one case I advised marriage with the assertion and positivism that the patient was immune, and, therefore, would never die from consumption. At some later day we will try the case again. A year may find our dictum set at naught; but it is in this manner, and by the enactment of laws regarding the public safety against the indiscriminate manner of destroying bacilli, that we must battle against this germ. Each patient will be taught that the germ is hard to destroy; that the disease can be communicated to their loved ones by the excreta, as sputa dried up and carried in the dust and air, and to prevent its destructive work, death to the germ is the only safeguard.

Every patient should be taught to use a clean piece of paper upon which to expectorate, and see that it is burned before it becomes dry. The day is not far distant when consumptives will be known, not by the cough and appearance, but by their carrying, strapped to their sides, a porcelain spittoon so arranged that the contents cannot be spilled; and, when convenient, conveyed to a furnace where the heat will be so great that the germs will be destroyed, and the spread thus prevented.

Dr. Von Ruck writes us that the operatives in his laboratories, where they work in and among the dried sputa, are subjected about twice a year to this immunizing process. So great is this blessing to the human race, that it is now possible to carry on an effective warfare against the tubercle bacilli. Only a few years have elapsed since the elucidation of this subject, yet the death rate from consumption is being reduced in all the civilized world, and no insurmountable obstacle now prevents the stamping out of this disease; and time and education will do it all.

With this brief outline before us, of the cause and effect, it becomes the duty of the profession to determine whether we are justified in the use of the antitoxines in tuberculosis. I believe that the immunizing substance is contained in the specific germ itself, as is shown by the success of immunization with the watery extract of tubercle bacilli. To my mind, the superiority of the pure tubercle bacillus substance in the form of watery extract is attested by unmistakably better results in clinical employment and in the animal experiments made by Dr. Von Ruck. The safety of the process, used in all stages of tubercular diseases without harm, and with results, as a rule, of the arrest of the disease, or with permanent cure, leads me to feel justified in adopting the idea and theories, and in giving it my hearty support, and encouraging those so diseased to hope that a cure can be promised. Dr. Von Ruck sends me his ideas on this line, and I herewith present it, as written by him, and trust that it will lead to discussion of same.

Dr. Osborn then read the following letter from Dr. Von Ruck:

